# Two birds with one stone: sustainable smart spectrophotometric methods for concurrent determination of silodosin and mirabegron: application to dosage forms and greenness assessment

**DOI:** 10.1186/s13065-025-01411-7

**Published:** 2025-03-03

**Authors:** Khalid M. Badr El-Din, Sayed M. Derayea, Ahmed S. Ahmed, Mohamed Oraby, Mohamed A. Abdelshakour

**Affiliations:** 1 Department of Analytical Chemistry, Faculty of Pharmacy, Minia University, Minia, 61519 Egypt; 2 Department of Pharmaceutical Analytical Chemistry, Faculty of Pharmacy, Sohag University, Sohag, 82524 Egypt; 3 Department of Pharmaceutical Chemistry, College of Pharmacy, Al-Esraa University, Baghdad, 10069 Iraq

**Keywords:** Silodosin, Mirabegron, UV- spectrophotometry, And pharmaceutical dosage form

## Abstract

A new combination of silodosin and mirabegron has recently obtained approval in the Indian market for addressing the benign prostatic hyperplasia symptoms associated with overactive bladder syndrome. In this study, we present four validated UV-spectrophotometric methods that rely on straightforward mathematical calculations for the quick and simultaneous assay of MRB and SLD in commercial tablets and synthetic mixes without the need for prior separation. The suggested methods include dual-wavelength, induced dual-wavelength, ratio difference, and area under the curve. These methods were effectively used to determine SLD and MRB simultaneously in combinations with severe spectrum overlap, showing excellent recoveries free from interference from pharmaceutical excipients. The proposed approaches were assessed and validated following the guidelines set forth by the International Conference for Harmonization (ICH). The methods exhibited linear ranges of 1–20 μg mL^−1^ and 1–25 μg mL^−1^ for SLD and MRB, respectively. Their environmental friendliness was assessed using the Analytical Greenness Calculator (AGREE) and The Green Analytical Procedure Index (GAPI) tools, demonstrating their supremacy in terms of greenness compared to the reported chromatographic method. There were no appreciable variations in accuracy or precision between the reported chromatographic method and statistical comparisons based on t- and F values. Consequently, these suggested methods are deemed effective in routine analysis of SLD and MRB, serving as cost-effective alternatives in quality control laboratories lacking expensive chromatographic instruments.

## Introduction

**Fig. 1:**
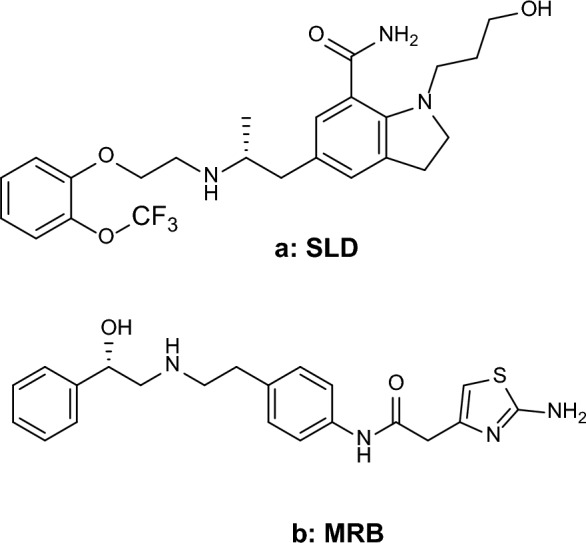
Chemical structure of (**a**): SLD and (**b**): MRB

Excessive activity of the bladder, a condition known as overactive bladder (OAB), is frequently noticed in males aged 45 and above, causing a substantial negative effect on their overall well-being. Healthcare providers commonly recommend alpha-blockers as a therapeutic approach to address OAB linked with benign prostatic hyperplasia (BPH) [1]. The severity and frequency of such symptoms vary significantly among individuals, encompassing a spectrum from urgency in the bladder, nocturia, and a feeling that the bladder is not completely emptying to sporadic force of the urine stream. These symptoms have the potential to cause lower urinary tract infections and possibly renal failure in extreme situations [2]. Recent findings suggest that combining antimuscarinics with alpha-1-blockers may offer a more effective solution for reducing BPH-related OAB compared to using alpha-blockers alone [3]. As a result, the pharmaceutical industry has focused its efforts on addressing both BPH and OAB disorders simultaneously. This involves the development of new drug combinations, such as the formulation containing silodosin (SLD) 8 mg and mirabegron (MRB) 25 or 50 mg in the Indian market [3–5]. In October 2008, the US Food and Drug Administration (FDA) approved SLD to be used in the treatment of benign prostatic hyperplasia (BPH). SLD functions as an antagonist for the alpha-1 adrenergic receptor [6]. SLD operates by decreasing the tone of contraction in the bladder neck and prostate smooth muscles, facilitating a smoother flow of urine. It is considered the preferred drug for treating BPH because, in comparison to other nonselective alpha-blockers, it has a lower frequency of systemic side effects [7, 8]. MRB holds the distinction of being the inaugural B3-adrenoceptor agonist approved by the FDA for the treatment of OBA. This approval was granted in 2012 [9]. MRB achieves its therapeutic effect by relaxing the smooth muscles of the detrusor during the urinary bladder's storage phase [10]. MRB's adverse effect profile is better than that of older antimuscarinic drugs, even though its effectiveness is equivalent [11]. The chemical name of SLD is 1-(3-hydroxypropyl)-5-[(2R)-2-([2-[2-(2,2,2-trifluoroethoxy)phenoxy]ethyl]amino)propyl]-2,3-dihydro-1H-indole-7-carboxamide Fig. 1 a. Many analytical approaches for the assay of SLD have been found in the literature. These methods include; spectrophotometric [12–15], spectrofluorimetric [16, 17], HPTLC [18–20], HPLC [21–23], UPLC [24, 25], and electrochemical methods [26–28]. The chemical name of MRB is 2-(2-amino-1,3-thiazol-4-yl)-*N* -[4-[2-[[(2*R*)-2-hydroxy-2-phenylethyl]amino]ethyl]phenyl]acetamide Fig. 1 b. Upon a review of the literature, it was found that many analytical methods for MRB have been reported. The methods were spectrophotometric [29–32], spectrofluorimetric [33–35], HPTLC [36, 37], HPLC UV [38–40], UPLC MS [41–43], and electrochemical methods [44]. Upon an extensive review of the literature, there has been only one reported technique for the concurrent measurement of SLD alongside MRB, which was the HPLC method [45].

While chromatographic methods offer the advantage of demonstrating exceptional sensitivity and can be utilized for investigating drug degradation products and pharmacokinetics, they are characterized by time-intensive processes. Moreover, their implementation demands expensive sophisticated instruments, substantial quantities of highly pure organic solvents, and a skilled workforce [45, 46].

Currently, the spectrophotometric technique is seen as an appropriate and sustainable choice in the field of instrumental analysis [47, 48]. The simplicity and time efficiency of this method, coupled with its minimal consumption of analytical solvents, render it a more cost-effective and environmentally friendly choice compared to alternative analytical approaches. Although UV-spectrophotometry often encounters challenges such as spectral overlaps when analyzing mixtures, this issue can be addressed through the application of algorithms for processing mathematical data after computation. Some of these algorithms are univariate, such as induced dual-wavelength approaches, and multivariate, such as partial least squares [49–51]. This motivated the authors to develop four spectrophotometric methods that are sensitive, cost-effective, and environmentally conscious, addressing the overlapping UV spectra of SLD and MRB without the requirement for previous separation by using simple mathematical computations. The suggested methods include dual-wavelength (DW), induced dual-wavelength (IDW), ratio difference (RD), and area under the curve (AUC) methods. Two eco-friendly assessment tools, namely the Analytical Greenness Calculator (AGREE) and The Green Analytical Procedure Index (GAPI), were utilized for environmental impact evaluation of the proposed approaches concerning the chemicals and procedures employed. The suggested approach has undergone thorough validation following the guidelines set forth by the International Council for Harmonization (ICH) [52].

## Experimental

### Instrumentation

Spectrophotometric measurements were conducted using UVWin 5 software in conjunction with a T80 double-beam UV–VIS spectrophotometer from PG Instruments in Leicestershire, UK. The data was acquired using quartz cells with a thickness of one centimeter. Double-distilled water from an Aquatron water still A4000D (Cole-Parmer, Staffordshire, UK) was used. Additionally, a Mettler Toledo 5-digit balance from Greifensee, Switzerland, was employed in the experiment.

### Materials and reagents

The standard for MRB with a confirmed purity of 99.5% was generously provided by Amoun Pharmaceuticals (El-Obour City, Egypt). Additionally, the standard for SLD, verified to have a purity of 99.6%, was graciously provided by Al Andalous Pharmaceutical Industries (the 6th of October City, Egypt). Sildocare^®^ tablets, indicating a stated SLD content of 8 mg, were procured from Al Andalous Pharmaceutical Industries. Flowadjust^®^ tablets, manufactured by Amoun Pharmaceuticals, labeled with a designated MRB content of 25 mg, were also obtained. Additionally, Bladogra^®^, manufactured by Apex Company (Cairo, Egypt), and claimed to have 50 mg of MRB, was bought from a local pharmacy.

Spectroscopic grade methanol and acetonitrile were provided by Merck in Darmstadt, Germany. Analytical grade sodium hydroxide was sourced from Fischer Scientific (Loughborough, UK). Analytical grade ethanol and hydrochloric acid were provided by El Nasr Pharmaceutical Chemicals Co. located in Cairo, Egypt.

### Preparation of standard solution

Stock standard solutions of SLD and MRB at a concentration of 250 μg mL^−1^ were set through the dissolution of 25 mg of each medication in 100 mL of methanol, and the solutions were then stored at 4 °C. Methanol was used to dilute the primary stock standard solutions in order to create working standard solutions.

### General assay procedures

Various portions of SLD and MRB were precisely transferred into two distinct sets of 10-mL volumetric flasks from each of the corresponding standard solutions (250 μg mL^−1^). These were then filled to their capacities with methanol to achieve concentration ranges of SLD (1–20) μg mL^−1^ and MRB (1–25) μg mL^−1^. Following this, the computer recorded the absorption spectra of SLD and MRB by scanning them over a 200–400 nm wavelength range against methanol as a blank. Every concentration of SLD and MRB was assayed in triplicate for accuracy.

#### DW method

To determine SLD in the presence of MRB, the values of absorbance were determined at 217.5 and 271 nm, and there was no absorbance difference for MRB at either wavelength. Conversely, to determine MRB in the presence of SLD, the values of absorbance were determined at 251 and 281 nm, and there was no absorbance difference for SLD at either wavelength. The absorption differences at the two chosen wavelengths for MRB and SLD were then plotted against the corresponding concentrations in the ranges of (2–25) μg mL^−1^ and (1–15) μg mL^−1^ to create calibration curves. Subsequently, regression equations were calculated for both SLD and MRB.

#### IDW method

To determine SLD in the presence of MRB, the absorption spectra of SLD at two specific wavelengths, 220 nm and 230 nm, were utilized for guidance in the selection process. Subsequently, the absorbance values of SLD at 230 nm were adjusted by multiplying them with the equality factor (F) for pure MRB, where F is equal to A220/A230 = 0.557. Conversely, to determine MRB in the presence of SLD, the absorption spectra of SLD at two specific wavelengths, 250 nm and 258 nm, were utilized for guidance in the selection process. Subsequently, the absorbance values of MRB at 258 nm were adjusted by multiplying them with the equality factor (F) for pure SLD, where F is equal to A250/A258 = 0.749. Subsequently, SLD and MRB's respective differences between the corrected absorbance values at 220 and 230 nm and the adjusted absorbance values at 250 and 258 nm were calculated. The difference of values at the specified wavelengths were then plotted against the concentrations of SLD (2–15 μg mL^−1^) and MRB (1–25 μg mL^−1^) to create calibration curves.

#### RD method

To determine SLD in the presence of MRB, the stored absorption spectra of SLD were divided by the spectrum of MRB at a concentration of 6 μg mL^−1^. Conversely, for the determination of MRB in the presence of SLD, MRB's zero-order absorption spectra were divided by the spectrum of SLD at a concentration of 4 μg mL^−1^. The amplitudes of the resulting ratio spectra were then measured at 222 and 245 nm for SLD. In addition, the ratio spectra were measured at 250 and 285 nm for MRB. To create the calibration curves, the differences between these amplitudes were then calculated. Plotting the wavelength-specific amplitude differences against the corresponding concentrations resulted in the calibration curves. For SLD, the concentration range was (2–20) μg mL^−1^ and was (2–25) μg mL^−1^ for MRB. At the end, the regression equations for SLD and MRB were calculated.

#### AUC method

SLD and MRB were calculated by measuring the AUC values for the absorption spectra of both medicines throughout the wavelength ranges of 217–223 nm (λ1–λ2) and 245–253 nm (λ3–λ4). Once the AUC values for SLD and MRB at the specified wavelength ranges were plotted against their corresponding concentrations, calibration curves were created. SLD and MRB had concentration ranges of (2–15) and (2–25) μg mL^–1^, respectively. The values of the area absorptivity constant (a) and regression equations for both medications were then calculated.

$${\text{Absorptivity values }}\left( {\text{a}} \right){\text{ of SLD or MRB}}\; = \;\frac{{{\text{AUC of SLD or MRB at }}(\lambda {1 } - \lambda {2}){\text{ or }}(\lambda {3 } - \lambda {4})}}{{\text{corresponding concentration of SLD or MRB}}}$$

### Assay procedure for synthetic mixtures

Five laboratory-mixed samples were prepared, each containing varying concentrations of SLD and MRB in various ratios (1: 1,1: 2, 2: 1, and the two available dosage form ratios 8: 25 and 8: 50). Various portions of SLD and MRB were precisely transferred into sets of 10 mL volumetric flasks from each of corresponding standard solutions (250 μg mL^−1^). These were then filled to their capacities with methanol to achieve the following concentrations: the first combination contained 4 μg mL^−1^ of SLD and MRB. The second combination contained 4 and 8 μg mL^−1^ of SLD and MRB, respectively, the third combination contained 4 and 12.5 μg mL^−1^ of SLD and MRB, respectively, the fourth combination contained 4 and 25 μg mL^−1^ of SLD and MRB, respectively, and the fifth combination contained 8 and 4 μg mL^−1^ of SLD and MRB, respectively. These samples were then assayed following the procedures outlined during the calibration curves development for the previously mentioned methods.

### Assay procedure for laboratory-made tablet formulation

In Egypt, pharmaceutical tablets combining SLD and MRB are not commercially available. To address this, a laboratory-made approach was adopted to simulate combination tablets. Twenty Sildocare^®^ tablets, each containing 8 mg of SLD, were weighed to calculate an average weight. Similarly, the weight of the contents of twenty MRB tablets (either Bladogra^®^ 50 mg or Flowadjust^®^ 25 mg) was determined to calculate an average weight. 70 ml of methanol were used to dissolve a quantity of powdered tablet equal to 40 mg SLD and 125 or 250 mg MRB. Following that, the mixture was carefully stirred in a 100 ml volumetric flask. After 15 min of sonication, the volume in the flask was adjusted by adding the appropriate amount of methanol. After that, the mixture was filtered, and the first part of the filtrate was disposed of. A portion of the filtrate underwent the assay procedures, and this process was repeated five times for replication.

### Evaluation methods of method greenness

To assess the eco-friendliness of the suggested spectrophotometric methods, two evaluation methods were employed: GAPI [53] and AGREE [54]. GAPI is a greenness evaluation method that considers fifteen parameters, spanning from sample collection to final analysis. The GAPI framework is represented by five pentagrams, each divided into three or four segments. A higher number of green segments indicates an eco-friendly process. The AGREE tool offers a novel method for assessing greenness. It uses a circular diagram divided into 12 segments, each representing one of the 12 principles of green analytical chemistry (GAC). This diagram is generated by easily accessible software available for download. The precise data provided by the AGREE program allows analysts to assign weights to the parameters based on their preferences.

## Results and discussion

**Fig. 2:**
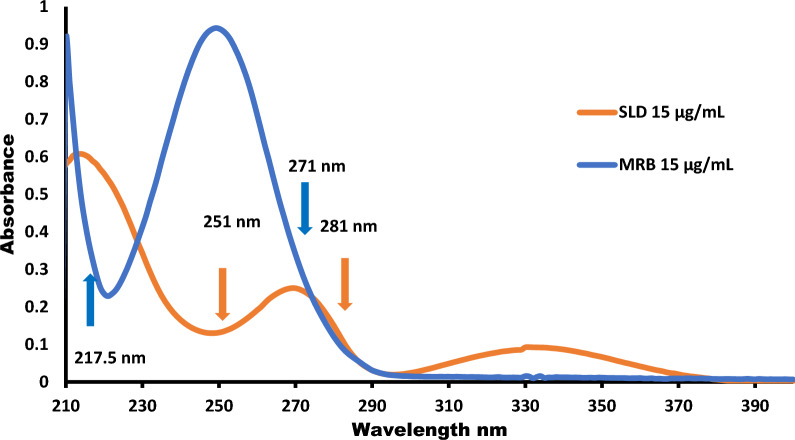
Overly zero absorption spectra of 15 μg mL^–1^ SLD (orange colour line) and 15 μg mL^–1^ MRB (blue colour line); showing the determination of SLD at (217.5–271) nm while MRB at (251–281) nm by the dual-wavelength method

The typical UV absorption profiles of SLD and MRB exhibit significant overlap, as illustrated in Fig. 2. This overlap poses challenges for the direct simultaneous determination of both substances. Therefore, four intelligent sustainable spectrophotometric methods, based on straightforward mathematical computations, were employed for the swift and simultaneous assessment of SLD and MRB in synthetic mixtures and a tablet formulation created in the laboratory. No preliminary separation was necessary; instead, straightforward mathematical manipulations of the spectra of the studied drugs were employed. This method does not demand specialized equipment or extensive training, making it a reliable option for routine analysis.

### Selection of diluting solvent

**Fig. 3:**
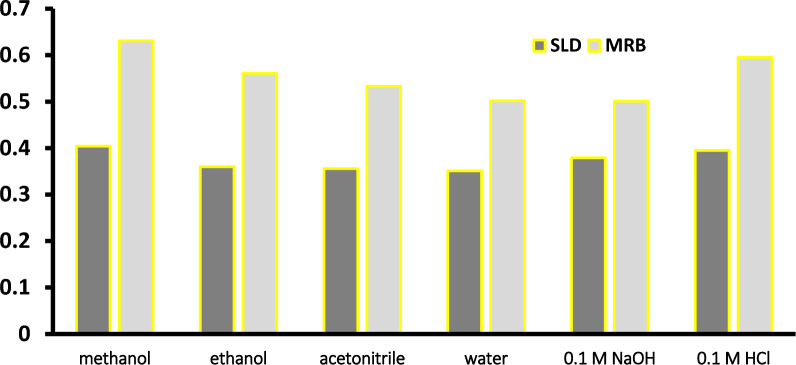
Effect of the diluting solvent on the proposed chemometric methods intensity (Both SLD and MRB are 10 μg mL^–1^)

The potential of many solvents as diluting agents was evaluated, as shown in Fig. 3. These solvents included methanol, water, ethanol, acetonitrile, 0.1 M NaOH, and 0.1 M HCl. Methanol demonstrated the highest absorbance among these options. As a result, methanol was chosen as the most suitable diluent.

### Optimization of the methods

#### DW method

DW method provides a straightforward and efficient way to eliminate interference between SLD and MRB in their combined mixtures, Without the need for specialized software or additional data processing [47]. To address this interference, two carefully selected wavelengths (λ1 and λ2) were utilized based on the spectra of the mixed drugs. This selection ensured that the absorbance difference between λ1 and λ2 was directly proportional to the concentration of the first drug, while this difference remained zero for the second interfering drug. For SLD determination, the wavelengths chosen were 217.5 and 271 nm, while for MRB determination, 251 and 281 nm were used (Fig. 2). The absorbance differences at the specified wavelengths were plotted against the concentrations of SLD and MRB to develop their respective regression equations, which were then used to compute the concentrations of each in their mixtures. For SLD, the concentration ranges were 1–15 μg mL^−1^, while for MRB, they were 2–25 μg mL^−1^.

#### IDW method

IDW method is a straightforward spectrophotometric technique utilized to overcome the significant overlay in the absorption spectra of two substances mixture when the interfering substance lacks points of equal absorbance at particular wavelengths [31]. By carefully choosing two wavelengths, 220 nm and 230 nm, to avoid MRB interference, this approach facilitates the determination of SLD in its mixing with MRB. This is accomplished by multiplying the equality factor (F) for the pure MRB (A220/A230 = 0.557) by the absorbance at 230 nm. Similarly, by choosing two wavelengths − 250 nm and 258 nm- to exclude SLD interference, MRB in its combination with SLD may be determined. This involves multiplying the equality factor (F) for the SLD (A250/ A258 = 0.749) by the absorbance at 258 nm. In order to equalize the absorbances of the interfering medication at the designated wavelengths where the other drug absorbances diverge, the equality factor (F) in this approach is essential. Therefore, the difference (multiplied by F = 0.557) between the corrected absorbance at 230 nm and the absorbance at 220 nm for SLD in the absorption spectrum of the drug mixture is related to SLD alone, with MRB interference canceled. Similarly, the difference between the absorbance at 250 nm for MRB and the corrected absorption at 258 nm (multiplied by F = 0.749) in the absorbance spectrum of the drug mixture is related to MRB alone, with SLD interference canceled. The equations provided below offer further clarification for implementing this method to assay SLD when it is mixed with MRB:

1 $$\Delta {\text{A }}\left( {{\text{A}}_{{1}} {-}{\text{ F}}_{{{\text{MRB}}}} {\text{A}}_{{2}} } \right)\; = {\text{ A}}_{{{\text{SLD1}}}} {-} \, \left( {{\text{F}}_{{{\text{MRB}}}} .\;{\text{A}}_{{{\text{SLD2}}}} } \right)$$

In this context, A_1_ denotes the absorbance of the mixture at a specific wavelength λ_1_ (220 nm), and A_2_ represents the absorbance at a different wavelength, λ_2_ (230 nm). F_MRB_ signifies the equality factor associated with pure MRB. According to Eq. (1), it becomes evident that the absorbance difference (A_1_–F_MRB_A_2_) in the mixture of the investigated drugs is solely influenced by the absorbance values of SLD (A_SLD 1_ and A_SLD 2_), without any dependence on the absorbance values of MRB.

2 $$\Delta {\text{A }}\left( {{\text{A}}_{{1}} {-}{\text{ F}}_{{{\text{MRB}}}} {\text{A2}}} \right)\; = \;\left( {{\text{slope }}.{\text{ C}}_{{{\text{SLD}}}} } \right) \, \pm {\text{ intercept}}$$

Therefore, Eq. (2) was utilized to determine the concentration of SLD, established through the plotting of absorption differences from pure SLD's absorbance spectra at 220 nm and 230 nm versus the corresponding concentrations within the range of 2 to 15 μg mL^−1^. Simultaneously, MRB concentrations, spanning from 1 to 25 μg mL^−1^, were calculated at 250 nm and 258 nm. The technique for MRB determination mirrored that of SLD, incorporating the use of the SLD's equality factor at identical wavelengths.

#### RD method

**Fig. 4:**
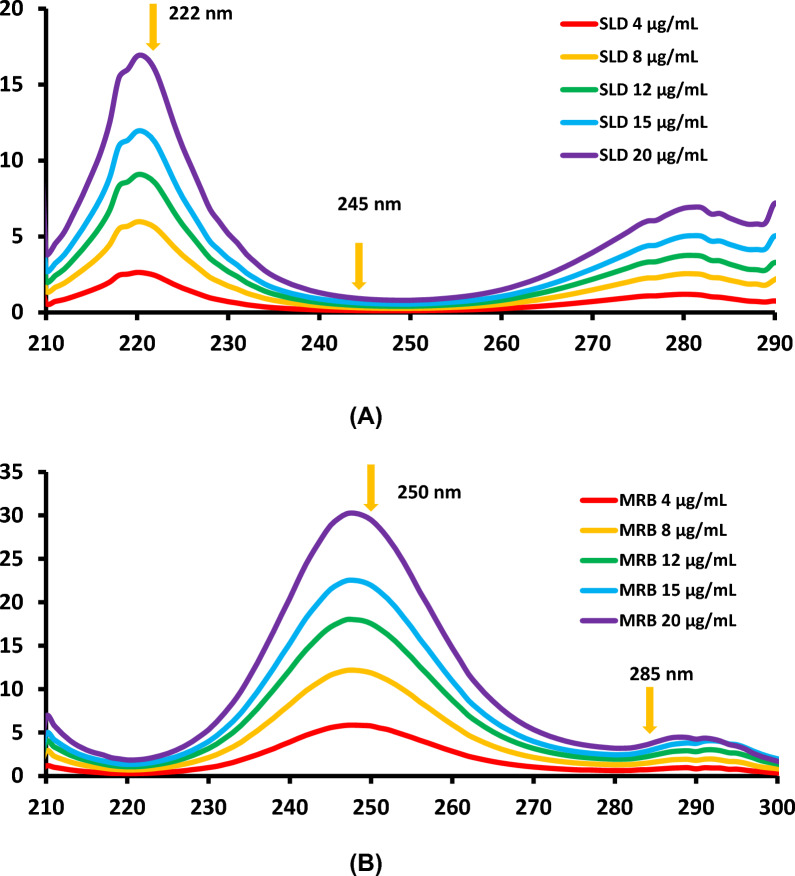
**A** Ratio difference spectra of SLD (4–20 μg mL^–1^) using MRB (6 μg mL^–1^) as a divisor; showing the determination of SLD at (222–245) nm and (**B**) ratio difference spectra of MRB (4–20 μg mL^–1^) using SLD (4 μg mL-1) as a divisor; showing the determination of MRB at (250–285) nm

RD technique, a straightforward computational method, is employed to eliminate SLD and MRB interference in their mixes without the necessity for separation using straightforward mathematical computations [31]. This method hinges on selecting wavelengths for computations that provide appropriate linear relationships as well as divisors with the least amount of noise and greatest sensitivity. By dividing their combination by an MRB divisor (6 g mL^−1^), after experimentation, SLD was successfully measured in its combination with MRB utilizing the RD technique (refer to Fig. 4a). The resultant ratio spectra amplitudes were determined at 222 and 245 nm and after that the computation of amplitude differences in order to eliminate MRB interference. Conversely, MRB in its combination with SLD was calculated by dividing it by SLD's divisor (4 g mL^−1^) (refer to Fig. 4b). The resultant ratio spectra amplitudes were determined at 250 and 285 nm, and amplitude differences were computed in order to eliminate SLD interference. The absorbance differences at the specified wavelengths were plotted against the concentrations of SLD and MRB to develop their respective regression equations, which were then used to compute the concentrations of each in their mixtures. For SLD, the concentration ranges were 2–20 μg mL^−1^, while for MRB, they were 2–25 μg mL^−1^.

#### AUC method

**Fig. 5:**
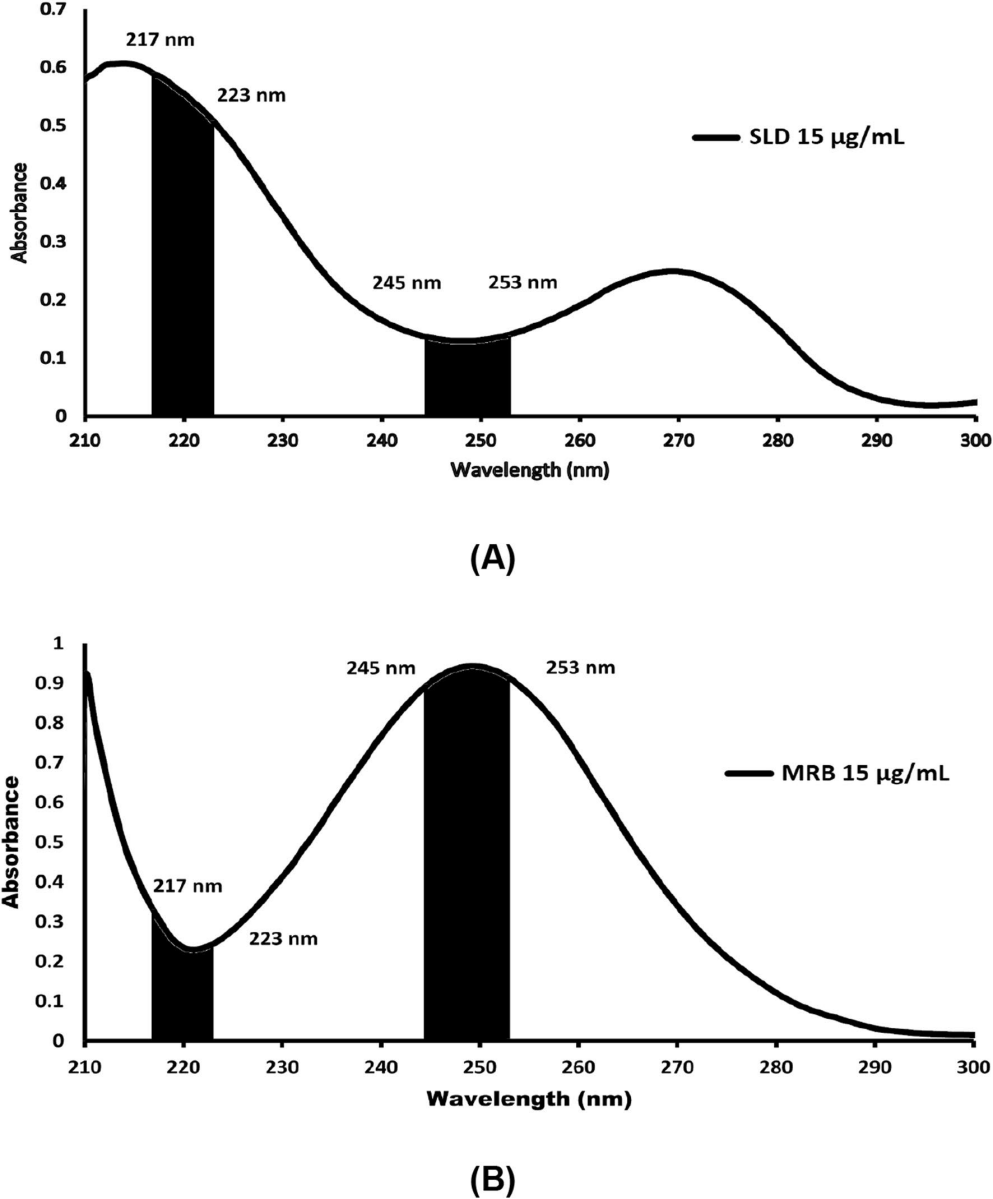
Areas under the curve over the wavelength ranges (217–223) nm and (245–253) nm of (**A**) zero-order absorption spectrum of SLD (15 μg mL^–1^) and (**B**) zero-order absorption spectrum of MRB (15 μg mL^–1^)

Without the need for specialized software or extra data processing, the AUC approach is distinguished by its speed and ease of use for minimizing interference between SLD and MRB in their mixed mixes [31]. This technique primarily consists of carefully selecting two pairings of wavelength ranges on the investigated medications' absorption spectrum, where concentrations may be calculated with straightforward mathematical operations [55]. In order to determine which wavelength range pairings would yield the best linearity correlations, a number of pairs were examined. The pairings that were ultimately chosen were 217–223 nm (λ1–λ2) and 245–253 nm (λ3–λ4), as depicted in Fig. 5 a and b.

After choosing these pairs, the AUC values for SLD and MRB at the specified wavelength ranges were plotted against their corresponding concentrations, ranging from 2 to 15 μg mL^−1^ for SLD and 2 to 25 μg mL^−1^ for MRB to create regression equations. The concentrations of SLD and MRB in respective mixes were then calculated using mathematical calculations based on the absorptivity constant (a) and AUC values at the specified wavelength ranges. The subsequent equations illustrate this computation process:

$${\text{C}}_{{{\text{SLD}}}} \; = \;\frac{{\left( {{\text{a}}_{{{\text{MRB }}(\lambda {1 } - \, \lambda {2})}} {\text{Auc}}_{{(\lambda {3 } - \, \lambda {4})}} } \right) \, - \, \left( {{\text{a}}_{{{\text{MRB }}(\lambda {3 } - \, \lambda {4})}} {\text{Auc}}_{{(\lambda {1 } - \, \lambda {2})}} } \right)}}{{\left( {{\text{a}}_{{{\text{MRB }}(\lambda {1 } - \, \lambda {2})}} {\text{ a}}_{{{\text{SLD }}(\lambda {3 } - \, \lambda {4})}} } \right) \, - \, \left( {{\text{a}}_{{{\text{MRB }}(\lambda {3 } - \, \lambda {4})}} {\text{ a}}_{{{\text{SLD }}(\lambda {1 } - \, \lambda {2})}} } \right)}}$$

$${\text{C}}_{{{\text{MRB}}}} = \;\frac{{\left( {{\text{a}}_{{{\text{SLD }}(\lambda {1 } - \, \lambda {2})}} {\text{Auc}}_{{(\lambda {3 } - \, \lambda {4})}} } \right) \, - \, \left( {{\text{a}}_{{{\text{SLD }}(\lambda {3 } - \, \lambda {4})}} {\text{Auc}}_{{(\lambda {1 } - \, \lambda {2})}} } \right)}}{{\left( {{\text{a}}_{{{\text{SLD }}(\lambda {1 } - \, \lambda {2})}} {\text{ a}}_{{{\text{MRB }}(\lambda {3 } - \, \lambda {4})}} } \right) \, - \, \left( {{\text{a}}_{{{\text{SLD }}(\lambda {3 } - \, \lambda {4})}} {\text{ a}}_{{{\text{MRB}}(\lambda {1 } - \, \lambda {2}) \, }} } \right)}}$$

In the context of the equations, C_SLD_ and C_MRB_ are the SLD and MRB concentrations expressed in μg mL^−1^. The terms a_SLD (λ1− λ2)_ and a_SLD (λ3− λ4)_ correspond to the absorptivity constant values of SLD at the wavelength ranges (217–223) nm and (245–253) nm, respectively. Similarly, a_MRB (λ1− λ2)_ and a_MRB (λ3− λ4)_ denote the absorptivity constant values of MRB at the specified wavelength ranges. The variables Auc _(λ1− λ2)_ and Auc _(λ3− λ4)_ represent the AUC values within their combination for the medications under study at (217–223) nm and (245–253) nm, respectively.

### Method validation

The proposed spectrophotometric techniques underwent an assessment and validation following the guidelines set forth by the ICH [52].

#### Linearity and range

In the specified experimental conditions, the assessment of the linearity of the proposed methodologies involved the examination of seven concentrations each for SLD and MRB. The concentration ranges were set at (1–20) μg mL^−1^ for SLD and (1–25) μg mL^−1^ for MRB. Regression analysis was conducted, and the results presented in Table 1 demonstrated a linear relationship between the concentrations of SLD and MRB within these ranges.

**Table 1:** The regression and validation parameters for the proposed spectrophotometric methods

Parameter	DW		IDW		RD		AUC			
	SLD	MRB	SLD	MRB	SLD	MRB	SLD (217–223)	SLD (245–253)	MRB (217–223)	MRB (245–253)
Linear range (µg mL ^−1^)	1–15	2–25	2–15	1–25	2–20	2–25	2–15	2–15	2–25	2–25
Slope	0.024	0.054	0.025	0.023	0.7398	1.2566	0.2726	0.0881	0.1189	0.5471
SD of slope (S_b_)	0.0002	0.0005	0.0004	0.0002	0.0091	0.0133	0.0032	0.0008	0.0013	0.0061
Intercept	− 0.0198	0.0056	− 0.0161	0.0022	− 0.7199	0.2577	− 0.2169	− 0.0912	− 0.0165	0.0559
SD of intercept (S_a_)	0.0016	0.0063	0.0033	0.0022	0.0958	0.1696	0.0294	0.0078	0.0174	0.0820
Correlation Coefficient	0.9998	0.9997	0.9995	0.9998	0.9995	0.9995	0.9997	0.9998	0.9996	0.9996
SD of residuals (S_y, x_)	3.5 × 10^–5^	0.0011	7.9 × 10^–5^	1.2 × 10^–4^	0.1833	0.7897	0.0064	0.0004	0.0054	0.1203
LOD (µg mL ^−1^)	0.22	0.39	0.44	0.32	0.43	0.45	0.36	0.29	0.48	0.49
LOQ (µg mL^−1^)	0.67	1.17	1.32	0.96	1.29	1.35	1.08	0.98	1.46	1.50

#### Limit of detection (LOD) and limit of quantification (LOQ)

The methods' sensitivity was assessed by computing the LOD and LOQ. Following ICH recommendations, the LOD was calculated as 3.3 times the standard deviation of the intercept divided by the slope, while the LOQ was determined as 10 times the standard deviation of the intercept divided by the slope. The obtained LOD and LOQ values are presented in Table 1.

#### Accuracy and precision

By calculating the mean percent recovery, the method's accuracy was assessed. The standard addition procedure was used to a sample from a previously analyzed laboratory-made combination. This approach involved adding three different concentrations of SLD and MRB standard solutions to the sample. Each concentration underwent triplicate analysis, and the percent recovery was calculated. Comprehensive outcomes are provided in Table 2. To evaluate the proposed method precision, three concentrations spanning the drugs' linearity ranges were determined. These analyses were conducted in triplicate, with assessments performed within a single day for intra-day precision and over three successive days for inter-day precision. As provided in Table 3, The results show that the tested drug's relative standard deviations continuously maintained below 2%, indicating great accuracy in both intra-day and inter-day measurements.

**Table 2:** Accuracy of the proposed spectrophotometric methods using standard addition method

Amount taken (μg mL^−1^)		Amount added (μg mL^−1^)		Amount found (μg mL^−1^)		% recovery ± SD	
SLD	MRB	SLD	MRB	SLD	MRB	SLD	MRB
DW							
2.00	4.00	0.00	0.00	2.00	3.98	100.05 ± 0.94	99.50 ± 0.45
2.00	4.00	1.00	2.00	3.01	5.90	100.40 ± 0.58	98.39 ± 0.22
2.00	4.00	4.00	8.00	6.05	12.11	100.84 ± 0.45	100.89 ± 0.29
2.00	4.00	13.00	21.00	15.08	24.86	100.55 ± 0.28	99.44 ± 0.26
IDW							
2.00	4.00	0.00	0.00	2.00	4.00	99.89 ± 0.49	100.11 ± 0.29
2.00	4.00	1.00	2.00	2.99	5.93	99.57 ± 1.08	98.79 ± 1.21
2.00	4.00	4.00	8.00	6.02	12.04	100.34 ± 0.38	100.34 ± 0.77
2.00	4.00	13.00	21.00	15.13	25.25	100.87 ± 0.56	101.00 ± 0.36
RD							
2.00	4.00	0.00	0.00	2.00	4.02	100.23 ± 0.68	100.87 ± 0.54
2.00	4.00	1.00	2.00	2.99	5.95	99.54 ± 0.69	99.09 ± 0.54
2.00	4.00	4.00	8.00	6.00	12.10	99.96 ± 0.96	100.86 ± 0.88
2.00	4.00	13.00	21.00	15.02	25.12	100.13 ± 0.59	100.48 ± 0.58
AUC							
2.00	4.00	0.00	0.00	1.97	3.99	98.70 ± 0.73	99.67 ± 1.30
2.00	4.00	1.00	2.00	2.99	5.92	99.57 ± 0.46	98.61 ± 0.59
2.00	4.00	4.00	8.00	6.08	11.90	101.37 ± 0.49	99.19 ± 0.49
2.00	4.00	13.00	21.00	14.82	24.93	98.83 ± 0.71	99.72 ± 0.42

**Table 3:** Evaluation of the intra- and inter-day precision for the proposed spectrophotometric methods

Method	Conc. level (μg mL^−1^)		%Recovery ± RSD			
	SLD	MRB	SLD	MRB	SLD	MRB
			Intra-day precision		Inter-day inter-day	
DW	2.0	4.0	98.87 ± 1.11	100.30 ± 0.36	99.80 ± 1.32	99.89 ± 0.55
	6.0	12.0	101.00 ± 0.70	101.06 ± 0.16	100.49 ± 1.05	100.88 ± 0.49
	15.0	25.0	100.23 ± 0.79	100.12 ± 0.40	100.01 ± 1.09	100.10 ± 0.47
IDW	2.0	4.0	99.51 ± 0.51	99.62 ± 0.92	99.86 ± 1.13	99.62 ± 1.23
	6.0	12.0	99.96 ± 0.34	100.64 ± 0.43	100.60 ± 0.77	100.73 ± 0.62
	15.0	25.0	100.30 ± 0.36	100.63 ± 0.32	100.00 ± 1.01	99.73 ± 0.98
RD	2.0	4.0	100.37 ± 0.52	98.91 ± 0.84	100.21 ± 1.15	99.35 ± 1.22
	6.0	12.0	99.02 ± 0.33	101.00 ± 0.42	99.96 ± 0.85	101.29 ± 0.60
	15.0	25.0	99.13 ± 0.33	100.51 ± 0.59	99.71 ± 0.77	100.79 ± 1.10
AUC	2.0	4.0	99.91 ± 1.36	99.94 ± 0.86	99.12 ± 1.36	99.51 ± 1.05
	6.0	12.0	100.58 ± 0.44	99.75 ± 0.76	100.79 ± 0.92	99.70 ± 0.66
	15.0	25.0	100.66 ± 0.53	100.62 ± 0.72	99.91 ± 1.20	100.11 ± 0.58

#### Selectivity

Five synthetic laboratory-prepared mixtures were analyzed in order to determine the proposed approaches' selectivity. These combinations had various SLD and MRB ratios, including the ratios found in their tablets, all of which were contained within the linearity limits of each compound (refer to Table 4). The %recoveries obtained from these analyses were deemed satisfactory, indicating the absence of interference in each combination between the medicines under study. This outcome confirms the proposed approaches' selectivity, as the presence of multiple components did not impact the accurate determination and recovery of the target substances in the mixtures.

**Table 4:** Evaluation of the selectivity for the proposed spectrophotometric methods

Conc. level (μg mL^−1^)		% recovery ± SD							
		DW		IDW		RD		AUC	
SLD	MRB	SLD	MRB	SLD	MRB	SLD	MRB	SLD	MRB
4.0	4.0	99.87 ± 0.71	101.18 ± 0.45	99.70 ± 1.17	99.95 ± 1.07	100.53 ± 0.96	100.63 ± 1.05	100.24 ± 1.12	101.11 ± 0.18
4.0	8.0	99.95 ± 0.74	100.80 ± 0.27	101.30 ± 0.35	100.00 ± 0.68	100.29 ± 0.73	101.28 ± 0.56	99.87 ± 0.67	101.17 ± 0.53
4.0	12.5	99.31 ± 1.17	100.06 ± 0.40	99.89 ± 0.93	100.62 ± 0.78	100.23 ± 0.64	100.08 ± 0.97	99.99 ± 0.52	101.28 ± 0.75
4.0	25.0	99.32 ± 0.63	100.40 ± 0.29	99.09 ± 1.14	100.29 ± 1.15	100.51 ± 0.69	100.74 ± 0.30	98.83 ± 0.89	101.29 ± 0.45
8.0	4.0	99.98 ± 0.46	100.24 ± 0.35	99.78 ± 0.62	99.87 ± 0.58	101.15 ± 0.88	99.88 ± 0.32	98.56 ± 0.69	101.66 ± 0.24

#### Pharmaceutical application

The investigation focused on assessing the applicability of the developed techniques in analyzing SLD and MRB within a laboratory-prepared tablet formulation. Table 5 presents favorable % recovery values obtained through the proposed methods, confirming that there is no matrix influence. To compare the results of the suggested approaches to a previously published technique, F- and student t-tests were also performed [45]. The calculated F- and student's *t*-test values were found to be below the tabulated values at the level of confidence of 95%, showing no discernible difference in precision and accuracy between the suggested and reported approaches. The findings support the assertion that the suggested method can accurately and precisely determine both SLD and MRB in combined tablets.

**Table 5:** Application of the proposed spectrophotometric methods to pharmaceutical formulation

Dosage form	Drugs	SLD				MRB					
	Parameters	DW	IDW	RD	AUC	Reported method^45^	DW	IDW	RD	AUC	Reported method^45^
Synthetic dosage form (SLD 8 mg and MRB 25 mg)	% recovery^a^	99.50	100.18	99.82	99.70	99.93	99.87	99.95	99.91	99.76	100.39
	SD	0.76	0.86	0.77	0.56	0.66	0.75	0.74	0.47	0.42	0.62
	t-value^b^	1.02	0.51	0.25	0.60		1.17	1.00	1.37	1.87	
	F-value^b^	1.03	1.68	1.37	1.42		1.44	1.40	1.79	2.23	
Synthetic dosage form (SLD 8 mg and MRB 50 mg)	% recovery^a^	100.35	100.51	99.58	99.99	100.31	100.67	100.17	100.54	100.79	100.38
	SD	0.48	0.54	0.60	0.67	0.75	0.36	0.71	0.70	0.49	0.58
	t-value^b^	0.11	0.49	1.69	0.71		0.96	0.52	0.41	1.22	
	F-value^b^	2.43	1.92	1.56	1.24		2.55	1.47	1.45	1.37	

^a^Mean of 5 determinations

^b^Tabulated value at 95% confidence limit; *t* = 2.306 and F = 6.338

#### Evaluation of method greenness

Analysts are essential in protecting people and the environment from dangerous substances and byproducts produced by sectors like as chemicals and pharmaceuticals [56, 57]. GAPI [53] and AGREE [54] approaches were employed to determine the greenness of an analytical procedure [58–60]. To assess the eco-friendliness of the suggested spectrophotometric methods, two evaluation instruments were employed: GAPI, and AGREE. To fully examine the ecological effect of different analytical approaches, it is advised to compare them using a variety of assessment tools. Also, they were compared with the reported chromatographic method.

GAPI provides qualitative data through pictogram symbols [53]. This figure represents a thorough assessment of the environmental effects of the analytical process. In order to assess specific aspects of the analytical procedure that can impact the environment, five pentagrams were made. The evaluation included three distinct color codes: red, yellow, and green, which represent minimal, medium, and severe environmental impacts, respectively. As shown in Table 6, the GAPI pentagrams indicate that the suggested procedures achieve a commendable green status, with 9 green, 4 yellow, and 2 red-shaded areas for the spectrophotometric methods, and 7 green, 4 yellow, and 4 red-shaded areas for chromatographic method.

**Table 6:** comparison between the proposed and reported methods

Item		Proposed method	Reported chromatographic method^45^
Linear range (μg mL ^−1^)		1–20 for SLD 1–25 for MRB	8–18 for SLD 24–54 for MRB
Application		Dosage form and greenness evaluation	Dosage form and stability Indicating
GAPI			
AGREE			
Greenness assessment parameters	Sample preparation		
	(1) Collection	Off-line	Off-line
	(2) Preservation	None	None
	(3) Transport	None	None
	(4) Storage	None	None
	(5) Type of method (direct or indirect)	Indirect (simple preparation)	Indirect (simple preparation)
	(6) Scale of extraction	Microextraction	Microextraction
	(7) Solvents/reagents used	Green solvents/reagents	Non-green solvents/reagents
	(8) Additional treatment	None	None
	Reagents and solvents		
	(9) Amount	< 10 mL	10–100 mL
	(10) Health hazard	Slightly toxic	Slightly toxic
	(11) Safety hazard	Safe	Safe
	Instrumentation		
	(12) Energy	less than 0.1 kW/h	≈ 0.5 kW/h
	(13) Occupational hazard	Hermetic sealing of the analytical process	Hermetic sealing of the analytical process
	(14) Waste	1–10 mL	> 10 mL
	(15) Waste treatment	No treatment	No treatment
	Quantification	Yes	Yes

AGREE, introduced recently for evaluating environmental sustainability [54], offers an unambiguous, flexible, and all-encompassing method. The calculator's software is easy to use, and deciphering its output is straightforward. The final outcome is displayed as a pictogram, with the sum of the scores in the center. The green color in the center of the image for both spectrophotometric and chromatographic procedures, as well as scores of 0.75 and 0.66, respectively, suggest that the spectrophotometric methods are highly green. The twelve GAC principles are also included into the pictogram; each is shown as a segment with a color code. Red denotes the least amount of greenness, while dark green indicates the greatest. The results of the AGREE for the suggested spectrophotometric and chromatographic procedures are shown in Table 6. The results from GAPI and AGREE confirmed that the suggested spectrophotometric methods were more environmentally friendly than the chromatographic approach.

### Comparison with a reported method

The suggested spectrophotometric techniques were evaluated alongside the chromatographic method previously mentioned, considering factors such as linear range, application, and the assessment of environmental sustainability. Table 6 presents a comparative examination of the proposed approaches with the chromatographic method. Notably, the proposed methods exhibit a relatively lower linear range compared to the chromatographic method. It is worth highlighting that the suggested methods are unique in their application to the evaluation of environmental friendliness, setting them apart from the previously reported approach.

### Comparison between the four suggested spectrophotometric techniques

The suggested spectrophotometric techniques differ in terms of their principles, linearity, sensitivity, and ease of application.

#### DW method

The DW method was highlighted for its simplicity and ability to eliminate interference from excipients. However, its performance may depend on the availability of suitable wavelength pairs with significant absorbance differences for the analytes of interest. The linearity range for SLD was 1–15 μg mL^−1^, while for MRB was 2–25 μg mL^−1^. The LODs were 0.22 and 0.39 μg mL^−1^ for SLD and MRB, respectively. While, the LOQs were 0.67 and 1.17 μg mL^−1^ for SLD and MRB, respectively.

#### IDW method

The IDW method provides improved selectivity by inducing mathematical wavelength manipulation. This method was advantageous in handling overlapping spectra but requires additional computational steps. The linearity range for SLD was 2–15 μg mL^−1^, while for MRB was 1–25 μg mL^−1^. The LODs were 0.44 and 0.32 μg mL^−1^ for SLD and MRB, respectively. While, the LOQs were 1.32 and 0.96 μg mL^−1^ for SLD and MRB, respectively.

#### RD method

The RD method offers a straightforward and selective approach, particularly when dealing with overlapping spectra. Its main advantage lies in its minimal reliance on complex instrumentation. However, it requires careful validation to ensure robustness across various sample matrices. The linearity range for SLD was 2–20 μg mL^−1^, while for MRB was 2–25 μg mL^−1^. The LODs were 0.43 and 0.45 μg mL^−1^ for SLD and MRB, respectively. While, the LOQs were 1.29 and 1.35 μg mL^−1^ for SLD and MRB, respectively.

#### AUC method

The AUC method relies on the integration of absorbance over a specific wavelength range, which is carefully selected to maximize the differentiation of analytes with overlapping spectra. However, it may require additional computational steps to calculate the integrated absorbance, making it slightly more time-intensive compared to simpler methods like DW or RD. The linearity range for SLD was 2–15 μg mL^−1^, while for MRB was 2–25 μg mL^−1^. The LODs were 0.29 and 0.48 μg mL^−1^ for SLD and MRB, respectively. While, the LOQs were 0.98 and 1.46 μg mL^−1^ for SLD and MRB, respectively.

## Conclusion

Based on the preceding discussion and findings, it can be deduced that the suggested methods were uncomplicated, cost-effective, responsive, and environmentally friendly for the simultaneous analysis of SLD and MRB without the need for initial separation. The proposed spectrophotometric methods exhibited linear ranges of 1–20 μg mL^−1^ and 1–25 μg mL^−1^ for SLD and MRB, respectively. The LODs ranged from 0.22 to 0.44 μg mL^−1^ for SLD and from 0.32 to 0.49 μg mL^−1^ for MRB. The LOQs ranged from 0.67 to 1.32 μg mL^−1^ for SLD and from 0.96 to 1.50 μg mL^−1^ for MRB. These methods were employed to concurrently assess SLD and MRB, displaying outstanding recovery rates in synthetic mixtures, and commercially available tablets, without being affected by the presence of pharmaceutical excipients. The synthetic dosage forms recoveries were found to be from 99.50 to 100.51% for SLD and from 99.76 to 100.79% for MRB. The proposed techniques exhibit a heightened level of environmental sustainability compared to the previously documented chromatographic method. The GAPI pentagrams indicate that the suggested procedures achieve a commendable green status, with 9 green, 4 yellow, and 2 red-shaded areas. The AGREE pictogram showed a score of 0.75, along with the green color at the middle of the picture. Consequently, these approaches are suitable for routine analysis of SLD and MRB in quality control laboratories that lack chromatographic instruments, as there were no significant statistical differences observed between these methods and the referenced chromatographic technique. The main limitation of the proposed spectrophotometric methods is disability to quantify cited drugs in biological fluids. So, the future aspect is to develop a more sensitive analytical method to determine them in human plasma and urine.

## Data Availability

All data generated or analyzed during this study are included in this published article.
